# Evaluation in Swine of a Recombinant Georgia 2010 African Swine Fever Virus Lacking the I8L Gene

**DOI:** 10.3390/v13010039

**Published:** 2020-12-29

**Authors:** Elizabeth Vuono, Elizabeth Ramirez-Medina, Sarah Pruitt, Ayushi Rai, Ediane Silva, Nallely Espinoza, James Zhu, Lauro Velazquez-Salinas, Douglas P. Gladue, Manuel V. Borca

**Affiliations:** 1 Agricultural Research Service (ARS), Plum Island Animal Disease Center, Greenport, NY 11944, USA; Elizabeth.Vuono@usda.gov (E.V.); Elizabeth.Ramirez@usda.gov (E.R.-M.); Sarah.Pruitt@usda.gov (S.P.); ayushi.rai@usda.gov (A.R.); Ediane.Silva@usda.gov (E.S.); Nallely.Espinoza@usda.gov (N.E.); James.Zhu@usda.gov (J.Z.); Lauro.Velazquez@usda.gov (L.V.-S.); 2 Department of Pathobiology and Population Medicine, Mississippi State University, P.O. Box 6100, Mississippi, MS 39762, USA; 3 Department of Pathobiology and Veterinary Science, University of Connecticut, Storrs, Mansfield, CT 06269, USA; 4 Oak Ridge Institute for Science and Education (ORISE), Oak Ridge, TN 37830, USA; 5 Department of Anatomy and Physiology, Kansas State University, Manhattan, KS 66506, USA

**Keywords:** ASF, ASFV, African swine fever, I8L gene, virus virulence, recombinant ASFV

## Abstract

African swine fever virus (ASFV) is the causative agent of African swine fever, a disease currently causing significant economic losses in Europe and Asia. Specifically, the highly virulent ASFV strain Georgia 2010 (ASFV-G) is producing disease outbreaks in this large geographical region. The ASFV genome encodes for over 150 genes, most of which are still not experimentally characterized. I8L is a highly conserved gene that has not been studied beyond its initial description as a virus ORF. Transcriptional analysis of swine macrophages infected with ASFV-G demonstrated that the I8L gene is transcribed early during the virus replication cycle. To assess the importance of I8L during ASFV-G replication in vitro and in vivo, as well as its role in virus virulence in domestic swine, we developed a recombinant virus lacking the I8L gene (ASFV-G-ΔI8L). Replication of ASFV-G-ΔI8L was similar to parental ASFV-G replication in primary swine macrophage cultures, suggesting that the I8L gene is not essential for ASFV-G replication in vitro. Similarly, replication of ASFV-G-ΔI8L in swine intramuscularly inoculated with 10^2^ HAD_50_ displayed replication kinetics similar to ASFV-G. In addition, animals inoculated with ASFV-G-ΔI8L presented with a clinical disease indistinguishable from that induced by the same dose of the virulent parental ASFV-G isolate. We conclude that deletion of the I8L gene from ASFV-G does not affect virus replication in vitro or in vivo, nor changes the disease outcome in swine.

## 1. Introduction

African swine fever virus (ASFV), the only member of the virus family *Asfarviridae*, is the etiological agent of African swine fever (ASF), a contagious disease currently affecting a large geographical area across central Europe, China, and South Asia [1,2]. This pandemic is responsible for substantial economic losses in the swine industry and worldwide protein availability shortages [2]. ASFV is a large, structurally complex virus with a large, double-stranded DNA genome of around 180–190 kilobases. The genome of the virus encodes for over 150 open reading frames (ORFs) with most still experimentally uncharacterized [1].

Currently, since there is no commercial vaccine available to prevent ASF; disease control relies on stopping regional animal movements, the instauration of proper biosecurity management, and culling affected animals [1,2]. Discovery of ASFV gene functions via genetic manipulation has enabled the production of experimental live-attenuated ASFV vaccine candidates [3,4,5,6]. Identifying and understanding viral genes that play a role in virus virulence is critical for the rational development of experimental vaccines using genetic manipulation. Therefore, identifying viral proteins that are important for in vitro and in vivo virus replication, and importantly in virus virulence in swine, is essential for the development of novel countermeasures to control the disease. Just a small number of genes have been successfully deleted from the ASFV genome, producing novel recombinant viruses (e.g., 9GL, UK, TK, MGF, NL, CD2, Lectin, DP148R, L83L, I177L, C962R, X69R) [3,4,5,7,8,9,10,11,12,13,14,15,16] with a subset of deleted genes determined to be essential for virus replication (e.g.: EP152R, p30, p54, and p72) [17,18,19,20]. Most ASFV proteins are characterized by predictive functional genomics analysis of ORFs, leaving much about individual ASFV genes and their translated products unknown [1,2].

Here we developed a recombinant ASFV with a deletion of the I8L gene (ASFV-G-ΔI8L) and assessed the effect of this modification on replication and virulence. We compared virus replication of ASFV-G-ΔI8L and its parental virus, demonstrating that the I8L gene is not essential for ASFV replication in vitro. We also demonstrated that deletion of the I8L gene from the genome of the ASFV Georgia 2010 (ASFV-G) isolate does not affect virus replication or virulence in swine, producing a clinical profile nearly identical to the virulent Georgia 2010 isolate. 

## 2. Materials and Methods

### 2.1. Cell Cultures and Viruses

Primary swine macrophage cultures were prepared from defibrinated blood, as previously described [15]. 

ASFV Georgia 2010 (ASFV-G) field isolate was kindly provided by the Laboratory of the Ministry of Agriculture (LMA) in Tbilisi, the Republic of Georgia by Dr. Nino Vepkhvadze [21].

Growth curves of ASFV-G-ΔI8L and parental ASFV-G were performed in primary swine macrophage cell cultures using 6-well plates. Cultures were infected at a MOI of 0.01 (based on HAD_50_ previously determined in primary swine macrophage cell cultures). After 1 h of adsorption at 37 °C under 5% CO_2_, the inoculum was removed, and the cells were rinsed two times with macrophage media and incubated with 2 mL of macrophage media at 37 °C under 5% CO_2_. At 2, 24, 48, 72, and 96 h post-infection (hpi), the cells were frozen at ≤−70 °C until the thawed lysates were used to determine titers by HAD_50_/mL in primary swine macrophage cell cultures. Virus titration was performed in 96-well plates of primary swine macrophages, the presence of virus was assessed by hemadsorption (HA), and virus titers were calculated as previously described [22].

### 2.2. Construction of the Recombinant Virus

**Figure 1:**
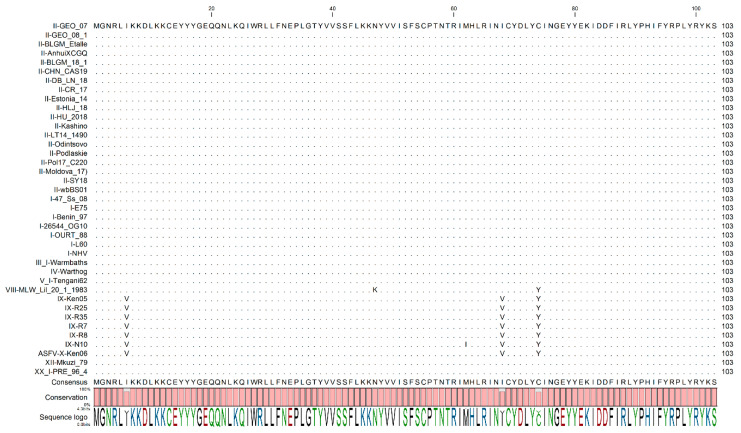
Multiple sequence alignment using CLC genomics workbench of I8L gene among the indicated African swine fever virus (ASFV) isolates for proteins. Matching residues are represented as dots. The degree of conservation is presented below the protein sequence and the conserved residue is presented on the bottom, indicating the degree of conservation for specific amino acid residues in the protein sequence.

Recombinant ASFV-G-ΔI8L was developed by homologous recombination between the parental ASFV genome and a recombination transfer vector, following standard procedures (Figure 1) [14,15]. The recombinant transfer vector (p72mCherryΔI8L) obtained by DNA synthesis (Epoch Life Sciences, Sugar Land, TX, United States), harbors two recombination arms flanking a reporter gene, mCherry, preceded by the ASFV p72 promoter [23]. The left and right recombination arms, covering 1000 bp on both sides of the I8L gene, are located between ASFV-G nucleotide positions 182,198–182,509, respectively; nucleotide positions are based on the reference genome ASFV Georgia 2007/1 (GenBank# LR743116) [24]. Macrophage cell cultures were infected with ASFV-G and transfected with the transfer vector. Infection and transfection efficiency were evaluated by the visual observation of mCherry fluorescence. Purification of the recombinant ASFV-G-ΔI8L was obtained by successive rounds of limiting dilution purification.

### 2.3. Microarray Analysis

Transcription of I8L gene was evaluated by microarray. The microarray data of the ASFV ORFs transcriptions were obtained from a previous study [25] published by us. The referred data is available at the Gene Expression Omnibus (GEO) repository, under the series record GPL26793. Background signal correction and data normalization of the microarray signals and statistical analysis were performed using the LIMMA package. The signal intensities of the ASFV ORF RNA were averaged from both Cy3 and Cy5 channels.

### 2.4. Complete Sequencing of ASFV Genomes Using Next-Generation Sequencing (NGS)

Viral DNA was extracted from macrophage cell cultures infected with ASFV-G-ΔI8L once the cytopathic effect was evident throughout the monolayer. DNA was extracted and then used to completely sequence the virus genome as previously described [23]. In brief, the viral DNA was sheared using enzymatic reactions assessed for the distribution of size fragmentation, then ligation of identifying barcodes using an adapter sequence was added to the DNA fragments. This DNA library was then used for next-generation sequencing (NGS) using the NextSeq 500 (Illumina, San Diego, CA, United States). Sequence analysis was performed using CLC Genomics Workbench version 20 software (CLC Bio, Waltham, MA, United States).

### 2.5. Animal Experiments 

Animal experiments were performed under BSL-3 conditions at the Plum Island Animal Disease Center (PIADC) facility, following a protocol approved by the (IACUC; 225.01-16-R_090716). ASFV-G-ΔI8L or parental ASFV-G were intramuscularly (IM) inoculated at a dose of 10^2^ HAD_50_ into a group of five 80–90-pound commercial breed swine. Clinical signs (anorexia, depression, fever, purple skin discoloration, staggering gait, diarrhea, shivering, and cough) and changes in body (rectal) temperature were recorded daily throughout the experiment.

## 3. Results and Discussion

### 3.1. I8L Gene Is Conserved across Different ASFV Isolates and Transcribed as an Early Gene

Sequence alignment of I8L from different ASFV genomes was performed using all genome sequences available at the Viral Bioinformatics Research Centre [26]. The multiple sequence alignment revealed a high degree of similarity of I8L among all isolates, with variation only at 5 residues; this variation was restricted to all genotype VII, IX, and X isolates (Figure 1).

Presence of I8L RNA transcripts was detected by DNA microarray analysis as we have previously reported [25]. I8L transcripts were detected at all time points. Expression gradually decreased from 3 to 9 hpi, then increased at 12 to 18 hpi. The transcriptional pattern was similar to the early protein p30 (CP204L) as previously reported [3]. Results demonstrated that the ASFV I8L gene encodes for a protein that is abundantly expressed early in the virus replication cycle.

### 3.2. Development of the ASFV-G-I8L Deletion Mutant

**Figure 2:**
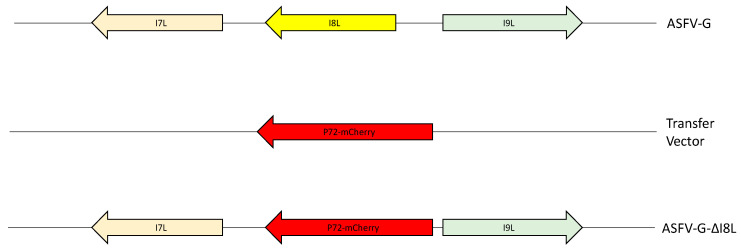
Schematic for the development of ASFV-G-ΔI8L. The transfer vector contains p72 promoter and the mCherry cassette, along with the flanking left and right arms of the transfer vector, designed to have flanking ends to both sides of the deletion/insertion cassette. The resulting ASFV-G-ΔI8L with the cassette inserted is shown on the bottom; the inserted cassette is a direct replacement for the ORF I8L.

To assess the function of the ASFV I8L protein in ASFV replication in macrophage cell cultures and domestic swine, a recombinant virus lacking the I8L gene was developed. The removal of the I8L gene from the ASFV Georgia isolate (ASFV-G) genome was performed by substituting the complete I8L ORF with the p72mCherry cassette by homologous recombination. The recombinant virus, ASFV-G-ΔI8L, was developed from the highly virulent ASFV-G. This genetic manipulation produced a 312-bp deletion (between nucleotide positions 182,198–182,509), completely removing the I8L ORF from the ASFV-G genome and replaced it with a 1226 bp cassette containing p72mCherry (see Materials and Methods) (Figure 2). The recombinant virus ASFV-G-ΔI8L was purified after 8 successive limiting dilution steps using primary swine macrophage cell cultures. ASFV-G-ΔI8L stocks were obtained by amplifying the virus population obtained from the last round of purification.

To assess genome integrity and confirm that the deletion of I8L was the only significant genomic modification incurred during the process of developing ASFV-G-ΔI8L. The full genome sequence of ASFV-G-ΔI8L was obtained by NGS on an Illumina NextSeq 500. Results confirmed the accuracy of the genomic modifications introduced and the absence of any additional significant mutations. NGS also confirmed the absence of any residual I8L gene from the parental ASFV-G genome contaminating the ASFV-G-ΔI8L stock. Importantly, the possibility of developing a purified population of ASFV-G-ΔI8L suggests that the I8L gene is not essential for the replication of ASFV-G in swine macrophages cultures. 

### 3.3. Replication of ASFV-G-*Δ*I8L in Primary Swine Macrophage Cultures

To assess the role of the I8L gene in virus replication, the in vitro characteristics of ASFV-G-ΔI8L were evaluated in primary swine macrophage cultures, the target cells infected during ASFV replication in swine, and compared to that of the parental ASFV-G in a multistep growth curve. Macrophage cultures were infected with the parental or the recombinant virus (MOI of 0.01), with presence of virus yield quantified at 2, 24, 48, 72, and 96 hpi. 

These experiments were performed using a low MOI to ensure that growth kinetics will require more than one replication cycle before the end of the experimental period. The additive effect of evaluating several successive replication cycles enhances the possibility of detecting subtle differences between the replicative abilities of these viruses, which may not be appreciated in a single step growth curve like that performed at a high MOI.

**Figure 3:**
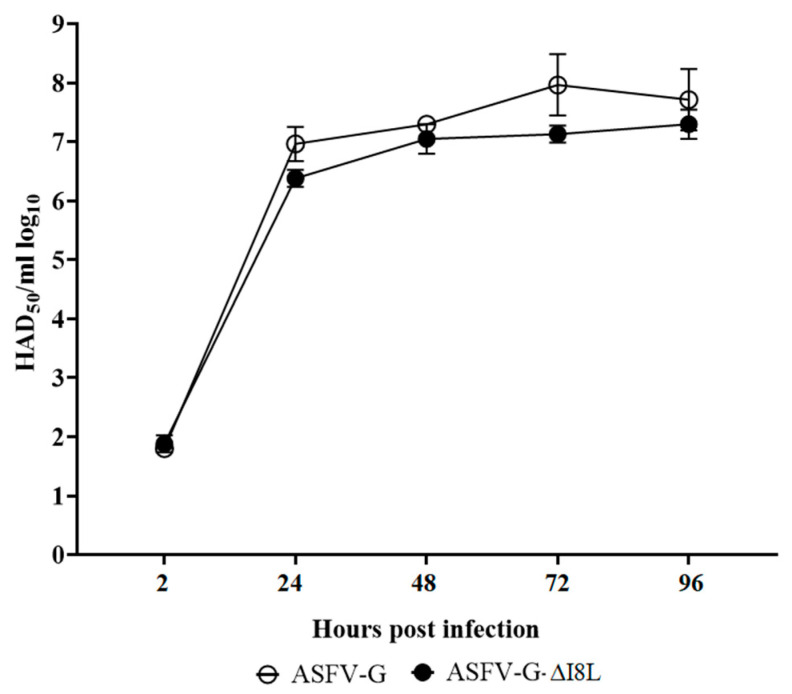
In vitro growth characteristics of ASFV-G-ΔI8L (filled symbols) and parental (empty symbols) ASFV-Georgia (ASFV-G). Primary swine macrophage cell cultures were infected (MOI = 0.01) with each of the viruses, and virus yield was titrated at the indicated times post-infection. Data represent means and SD from three independent experiments. Sensitivity of virus detection: >1.8 log_10_ HAD_50_/mL.

Results demonstrated that ASFV-G-ΔI8L displayed an almost identical growth kinetic to that of the parental ASFV-G (Figure 3). These results indicate that the deletion of the I8L gene does not significantly affect the ability of the virus to replicate in primary swine macrophage cultures. 

There is no information regarding the possible biological function(s) of the I8L gene, including virus replication. Mounting evidence suggests an increasing number of virus genes are not essential for ASFV replication [4,5,7,8,9,10,11,17,27]. It is possible that I8L gene function is duplicated by a different ASFV gene that is yet to be elucidated.

### 3.4. Assessment of ASFV-G-*Δ*I8L Virulence in Swine

Although there were no significant differences in replication of ASFV-G-ΔI8L in primary swine macrophage cultures when compared to the parental strain ASFV-G, it was important to determine if the recombinant virus efficiently replicates in vivo and produces disease as efficiently as the parental ASFV-G. The role of the I8L gene on the process of disease production by ASFV-G in domestic swine was assessed by infecting pigs with ASFV-G-ΔI8L, as described in Material and Methods section. Low virus doses were selected for the study, due to the very low 100% lethal dose of ASFV-G, to increase the probability of detecting subtle differences in virulence between ASFV-G-ΔI8L and the parental virus.

**Figure 4:**
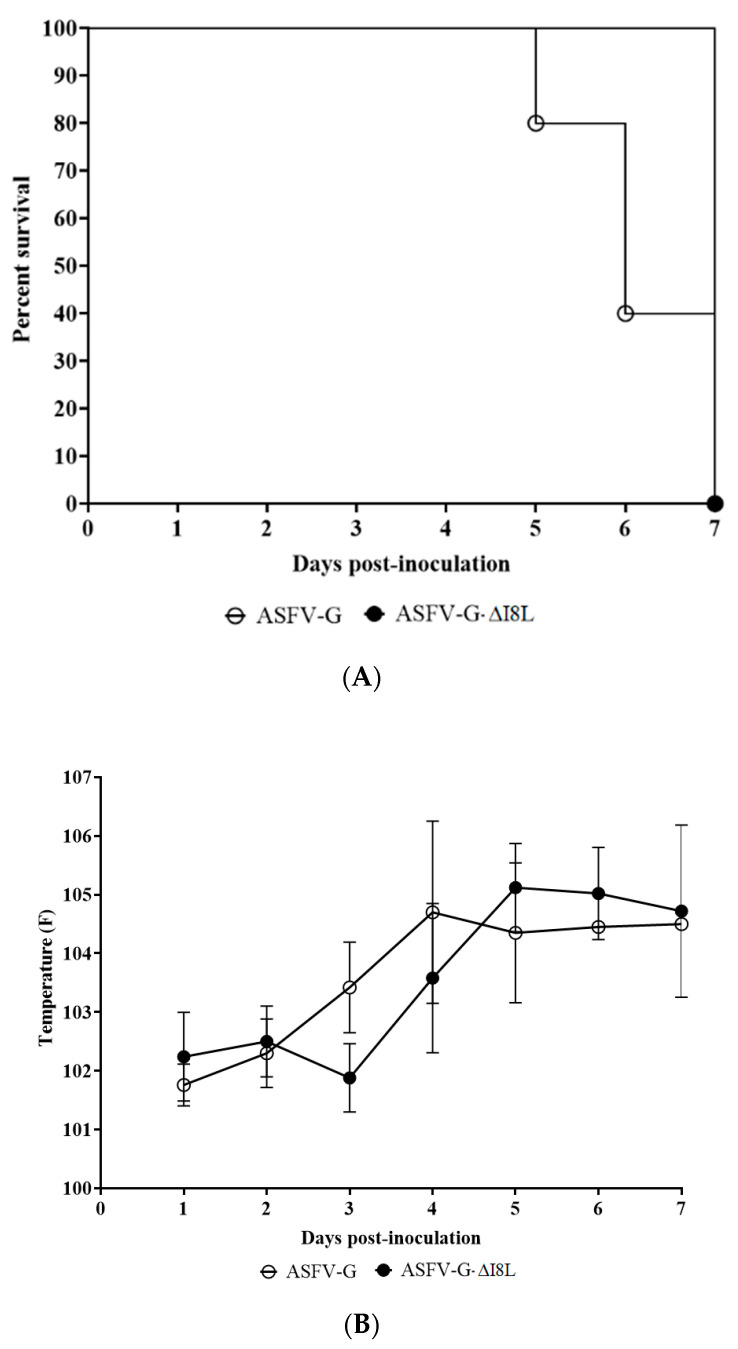
Progress of mortality (**A**) and body temperature (**B**) in animals intramuscularly (IM) infected with 10^2^ HAD_50_ of either ASFV-G-ΔI8L (filled symbols), or parental ASFV-G (open symbols). Panel B shows the average data and the corresponding SD.

Animals infected with ASFV-G exhibited typical onset of the disease, marked by an increase in body temperature (>104 °F) by day 4–5 post-infection, followed by the additional appearance of classical clinical signs associated with ASF (anorexia, depression, purple skin discoloration, vomit, diarrhea, and lastly, neurological signs) (Table 1 and Figure 4). The clinical disease was quickly aggravated, and animals were euthanized in extremis by day 5–7 post-infection. Animals receiving 10^2^ HAD_50_ of ASFV-G-ΔI8L experienced clinical disease nearly indistinguishable from those inoculated with ASFV-G, with disease onset by day 3–4 post-infection, the severity of disease evolving quickly, and animals being euthanized by day 7 post-infection. These observations suggest the deletion of I8L from the ASFV-G genome does not significantly alter its virulence in domestic pigs.

**Figure 5:**
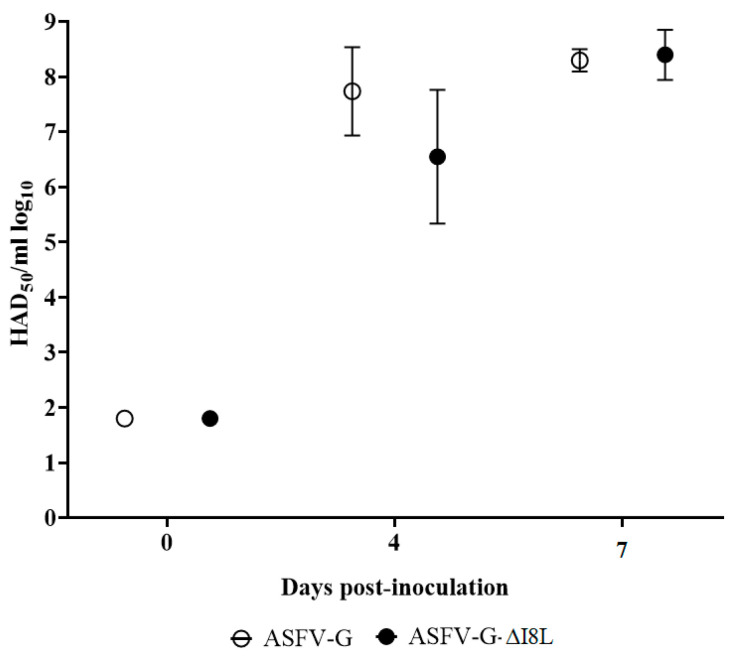
Viremia titers detected in pigs IM inoculated with 10^2^ HAD_50_ of either ASFV-G-ΔI8L (filled symbols) or ASFV-G (empty symbols). Each time point represents the average and SD of values in each of the groups. Sensitivity of virus detection: >log_10_ 1.8 HAD_50_/mL.

Viremia values in animals infected with ASFV-G were, as expected, very high (10^7^–10^8.5^ HAD_50_/mL) by day 4 post-infection, with titers remaining high until animals were euthanized at day 7 post-infection. ASFV-G-ΔI8L-infected animals also presented high viremias (values ranging from 10^5.5^ to 10^8^ HAD_50_/mL) by day 4 post-infection. Along with the progression of the disease, titers reached values similar to those of animals infected with ASFV-G by day 7 post-infection, before animals were humanely euthanized (Figure 5). This study demonstrated that virulence of ASFV-G-ΔI8L is indistinguishable from that of parental ASFV-G in domestic pigs.

In this study, we showed that I8L (a previously uncharacterized ASFV ORF), encodes a protein that is transiently expressed at early times during infection of swine primary macrophages. We also demonstrated that I8L is a non-essential gene since its deletion from the ASFV-G genome does not significantly alter virus replication in swine macrophage cultures. Importantly, the deletion of the I8L gene is not critical for ASFV virulence in swine, as the deletion mutant ASFV-G-ΔI8L had similar pathogenesis as the parental ASFV-G. Animals inoculated at very low doses (10^2^ HAD_50_) developed a disease indistinguishable from those receiving the parental fully virulent virus.

Interestingly, I8L was neither detected in the ASFV proteome [28], or as being part of the virus particle [29,30,31], complicating the understanding of the potential function of the gene. Surprisingly, the deletion of a gene does not alter (to some degree) the virus phenotype in terms of replication of disease production. Nevertheless, due to the extremely high virulence of the ASFV-G exhibiting such a low 100% lethal dose, it is conceivable that the deletion of one gene could not radically change the virulent phenotype. Perhaps the use of a natural route of infection (as infection by cohabitation with infected pigs), or performing deletion in a less virulent parental ASFV isolate will detect subtle changes in the virulent phenotype when the I8L gene is deleted. Further studies would be required to determine if this is the case. It is also possible that the function of I8L overlaps with other proteins in ASFV, and the simultaneous deletion of these additional unknown proteins would be required to give a more pronounced phenotype and fully disclose the functional role of I8L. It is possible that I8L gene may play a different role in ASFV isolates and the ASFV-G. It has been repeatedly shown that even highly conserved viral genes may have different functionality when analyzed in different isolates [5,16,32,33]. In addition, there is the possibility that I8L gene may play a critical role in any of the other ASFV natural hosts rather the domestic swine, wild swine, and soft ticks [2]. 

The lack of information on the essentiality of ASFV genes in either replication or virus virulence is a significant gap in knowledge for basic ASFV virology, which requires further research to understand the necessary components to cause disease. However, determining that the I8L gene can be deleted, and showing that it is non-essential in virus replication and disease production is an important step for determining the potential minimal essential genome for ASFV. Improving our current understanding of the proteins required for the pathogenesis of ASFV and the viral molecular mechanisms that occur during infection can allow for the construction of better rational vaccine designs.

## Figures and Tables

**Table 1:** Swine survival and fever response following infection with ASFV-G-ΔI8L and parental ASFV-G.

Fever					
Virus (10^2^ HAD_50_)	No. of Survivors/Total	Mean Time to Death (±SD)	No. of Days to Onset (±SD)	Duration No. of Days (±SD)	Maximum Daily Temp., F (±SD)
ASFV-G-ΔI8L	0/5	7 (0)	3.8 (0.84)	2.4 (0.55)	105.1 (0.79)
ASFV-G	0/5	6.2 (0.84)	4.6 (0.55)	2.4 (0.55)	104.7 (0.28)

## Data Availability

Not applicable.
